# Measuring motor intent for BCI control–A comparative analysis of signal quality of simultaneously recorded vECoG and scalp EEG

**DOI:** 10.1088/1741-2552/ae9eef

**Published:** 2026-09-17

**Authors:** Nikole Chetty, James Bennett, Hunter R Schone, Adam Fry, Peter E Yoo, David Lacomis, Thomas J Oxley, Jennifer L Collinger, David F Putrino, Douglas J Weber

**Affiliations:** 1 Department of Mechanical Engineering, Carnegie Mellon University, Pittsburgh, PA, United States of America; 2 Department of Medicine, University of Melbourne, Parkville, Victoria, Australia; 3 Synchron Inc., New York, NY, United States of America; 4 Rehab Neural Engineering Labs, University of Pittsburgh, Pittsburgh, PA, United States of America; 5 Department of Physical Medicine and Rehabilitation, University of Pittsburgh, Pittsburgh, PA, United States of America; 6 Department of Rehabilitation and Human Performance, Icahn School of Medicine at Mount Sinai, New York, NY, United States of America; 7 Departments of Neurology and Pathology (Neuropathology), University of Pittsburgh School of Medicine, Pittsburgh, PA, United States of America; 8 Department of Bioengineering, University of Pittsburgh, Pittsburgh, PA, United States of America; 9 Department of Biomedical Engineering, Carnegie Mellon University, Pittsburgh, PA, United States of America; 10 The Neuroscience Institute, Carnegie Mellon University, Pittsburgh, PA, United States of America

**Keywords:** brain-computer interface, vascular electrocorticography, electroencephalography, EEG, vECoG, stent-electrode array, at-home BCI systems

## Abstract

*Objective.* Stent-electrode arrays enable endovascular brain–computer interfaces (BCI) by recording cortical neural activity from within the superior sagittal sinus and have recently been evaluated in an early feasibility clinical trial in the United States (ClinicalTrials.gov: NCT05035823). For a BCI to be viable, the signals need to be high quality to enable accurate decoding of user intent. Compared to electrodes placed on the scalp for electroencephalography (EEG), stent-electrode arrays lie closer to the cortical surface and would presumably offer higher signal quality yet a direct comparison of intravascular and scalp-based neural recordings in humans has not yet been investigated. *Approach.* We directly compared the signal quality of vascular electrocorticography (vECoG) versus scalp EEG signals in one participant with severe upper limb paralysis due to ALS. During two experimental sessions, the participant underwent simultaneous recording with the stent-electrode array and a scalp EEG using a gel cap. The participant was visually cued to attempt motor tasks, such as repeated flexion and extension of the ankles. Signal quality was assessed by quantifying motor modulation strength, differentiation of movement effort, and spatial lateralization. Noise metrics evaluated the relative impact of artifacts including 60 Hz line noise, electrocardiogram contamination, eye blinks, jaw clenching, and vocalization. *Main results.* Both recording modalities exhibited significant modulation during attempted movement relative to rest, with vECoG generally demonstrating significantly stronger modulation per channel in some frequency bands and conditions. Motor modulation was significantly reduced during motor imagery compared to overt movement in both modalities. Spatial source localization between left and right ankle movement did not reach significance for either modality. Each modality was vulnerable to some artifacts while generally unaffected by others. Scalp EEG showed large susceptibility to ocular artifacts and cranial muscle activity due to its proximity to superficial physiological sources, whereas vECoG exhibited prominent cardiac activity. *Significance.* Within this participant, the large modulation during attempted movement recorded with vECoG, unaffected by the attenuating effects of the skull, coupled with fewer artifacts in the frequency bands of interest, provides preliminary evidence that the stent-electrode arrays can acquire high quality neural signals that could support BCI control.

## 1. Introduction

Brain–computer interfaces (BCIs) have the potential to increase independence for individuals with severe paralysis by enabling control of digital devices, facilitating communication and digital instrumental activities of daily living [1, 2]. Successful BCI functionality depends in part on the quality of recorded signals, with higher quality signals generally enabling more accurate and reliable performance.

Non-invasive BCIs, such as scalp electroencephalography (EEG), offer a non-invasive means of recording electrical biopotentials from the scalp. While used widely within research settings, scalp EEG performance for daily use is constrained by several factors, including low signal-to-noise ratio (SNR) and susceptibility to environmental and physiological artifacts. Furthermore, the skull and scalp tissues attenuate and spatially smear the neural signals due to volume conduction [3–8]. In addition, scalp EEG requires daily setup and often requires extensive training, making it less convenient and more burdensome for long-term, independent use in the home environment.

Stent-based electrode arrays are an emerging BCI technology that are implanted endovascularly. This approach involves a catheter-based implantation procedure, with current stent-electrode arrays providing targeted cortical recordings with a longitudinal coverage of approximately 25 mm. For example, with proper positioning within the superior sagittal sinus (SSS), the stent-electrode arrays can record bilaterally from the motor cortices and surrounding cortical areas without the need for a craniotomy. Recently, two publications have demonstrated successful decoding and BCI use in four individuals with severe bilateral upper extremity paralysis [9, 10]. Consistent with electrocorticography (ECoG) based BCIs, vascular ECoG (vECoG) BCIs leverage sensorimotor rhythms during both imagined and attempted movement, including beta band de-synchronization and, most prominently, high frequency neural features, such as gamma band synchronization, which provide rich and spatially focal information [11–17].

For any BCI to be viable, the signals must be high quality to enable accurate and reliable decoding of user intent. Multiple factors contribute to the SNR including distance to neural source, neuroanatomy, electrical contact and spacing, referencing scheme, physiological noise (such as ocular, muscular, cardiac, etc), environmental noise, and task attention. Compared to electrodes placed on the scalp for EEG which lie approximately 14–20 mm from cortex, the central axis of stent-electrode arrays in the SSS lie on average 6.1 mm from cortex, with electrodes on the stent scaffold adjacent to the cortex lying considerably closer [18–21]. However, vECoG currently remains constrained by the cortical venous system. Although endovascular BCIs such as the stent-electrode arrays are presumed to offer superior signal quality, greater bandwidth, greater spatial specificity, and lower susceptibility to artifacts by sitting closer to the cortical surface and avoiding the interceding bone, no human studies have directly compared intravascular and scalp-based recordings.

Here, we present the first direct, within-subject comparison of signal quality between simultaneous vECoG and scalp EEG recordings in a human participant. Neural activity was recorded with a stent-electrode array implanted in the SSS and from a commercially available EEG cap in a single-participant with paralysis due to ALS. We evaluated signal and noise characteristics across the standard frequency bands including both task-related neural modulation and baseline activity. Signal metrics included movement modulation strength, resolution of graded control signals, and spatial lateralization. Noise metrics captured spontaneous artifacts including 60 Hz line noise, electrocardiogram (ECG) contamination, and eye blink artifacts as well as prompted artifacts from jaw clenching and vocalization. Together, these analyses provide a comprehensive characterization of the relative advantages and limitations of vECoG and scalp EEG recordings with respect to signal quality and artifact susceptibility. As implanted BCIs approach clinical availability as a motor neuroprosthesis, such comparisons are essential for determining how, and to what extent, implanted BCIs perform in comparison to non-invasive alternatives.

## 2. Methods

### 2.1. Clinical trial and participant information

The study was approved by the WCG Institutional Review Board (1347924), with all study procedures conducted in accordance with the Declaration of Helsinki. The participant, C6, was consented to participate in the COMMAND Early Feasibility Study (clinicaltrials.gov NCT05035823), which sought to evaluate safety and feasibility of the endovascular motor neuroprosthesis to restore control of digital devices. The study was conducted under an Investigational Device Exemption (IDE G210178) granted by the US Food and Drug Administration. The data presented here were part of a limited set of experiments unrelated to the study’s prespecified primary outcomes.

The participant, C6, was a 63 year-old, white male diagnosed with amyotrophic lateral sclerosis (ALS) with bilateral flail arm syndrome (also known as man-in-barrel syndrome) approximately 2.5 years prior to implantation. The participant had been implanted with a stent-electrode array (Stentrode, Synchron, USA) that was deployed in the SSS adjacent to the motor cortices which has been described previously [9, 10, 17, 18]. Prior to implantation, the participant underwent structural and functional MRI scans to confirm the participant could activate cortical motor networks during executed movements [18]. Three months post-implant, CT scans identified the location of the stent-electrode array along the SSS.

At the time of recording, the participant was ambulatory and verbal. The participant had severe paralysis of the upper extremities but at baseline was able to produce visible movement of the digits over a limited range of motion. Manual muscle strength was assessed by a clinician using the Medical Research Council (MRC) scale. The MRC scale ranges from 0 (no visible muscle contraction) to 5 (normal strength with full range of motion against resistance). The following movements were assessed on both sides of the body: finger abduction (little finger), distal phalanx middle finger flexion, wrist extension, elbow flexion, elbow extension, hip flexion, knee extension, ankle dorsiflexion, ankle plantar flexion, big toe dorsiflexion. At the pre-implant baseline, the tested upper limb movements scored between 0 and 2 (mean 0.8). In contrast, the tested lower limb movements scored a 4 or 5 (mean 4.9). At 12 months post-implant, all tested upper limb movements scored 0, while lower limb movements scored a 4 or 5 (mean 4.6).

### 2.2. Simultaneous recordings

The participant underwent two sessions of simultaneous EEG and vECoG recordings. The sessions took place 268 and 475 d after implantation (training sessions 58 and 111). Each session lasted approximately 3 h, including setup and cleanup. The participant was seated comfortably in a chair with a laptop positioned on a table directly in front of him. His arms rested in his lap throughout the session. The laptop provided visual cues prompting the participant to perform a specific action. Data collection was split into a series of distinct runs, each typically lasting 2–5 min. The participant was asked to remain silent and limit extraneous movement during the runs. Between runs, the participant could rest, stretch, or communicate with the research team.

EEG signals were acquired using a 64-channel cap (Infinity Gel Headcap, TMSi) which was wired to the associated amplifier (SAGA, TMSi). The cap size was selected based on the participant’s head circumference. Electrodes (Ag/AgCl) were positioned according to the international 10–10 system with each electrode individually gelled (Signagel, ParkerLabs). No abrasive scalp or skin preparation was used prior to electrode placement. Conductive gel was applied to each scalp electrode using a blunt syringe and distributed with a small circular motion to improve electrode-skin contact. Adhesive electrodes were placed on the mastoids to provide reference and ground channels. For bipolar, reference, and ground electrodes, the skin was cleaned with an alcohol wipe prior to application of adhesive electrodes. The referencing scheme was a common average reference. Signals were sampled at 2000 Hz with an analog bandwidth of 700 Hz. Electrode impedances were generally below 30 kΩ, with lower impedances preferred where possible.

Scalp EEG electrode impedance was measured using a 100 mV peak-to-peak, 8 Hz square wave signal. Impedance measurements indicated 64.5% of electrodes were below <10 kΩ, while 35.5% were between 10–30 kΩ (supplemental figure 6). Preparation time and participant tolerability was prioritized over optimization of impedance at each electrode. vECoG signals were acquired using a first-generation, 16-channel stent-electrode array (Stentrode, Synchron, Investigational Device) whose lead was tunneled to a multichannel bioamplifier housed in an implantable receiver transmitter unit (IRTU) implanted in a subcutaneous pocket below the clavicle. Platinum electrodes were positioned at various nodes of the stent-strut. The reference electrode was either one of the 16 electrodes on the stent (referred to as local reference) or an electrode on the housing of the IRTU (referred to as IRTU reference). Signals were sampled at 2000 Hz with an analog bandwidth of 500 Hz. The signals were transmitted via infrared to the external receiver transmitter unit which was then sent to the signal control unit and then onto the recording laptop. Table 1 summarizes the key device specifications for the two recording systems.

**Table 1.** Device properties.

	vECoG (Stentrode, Synchron)	Scalp EEG (SAGA, TMSi)
Number of channels used	12	62
Sampling rate	2,000 Hz	2,000 Hz
Input referred noise*	2.4 *μ*V_rms_	< 1 *μ*V_rms_
Bandwidth*	500 Hz	700 Hz
Minimum electrode spacing (approximate)	0.37 cm	3.4 cm

* Based on data sheets for amplifier

Additional electrophysiology signals were acquired using bipolar leads with disposable snap electrodes, acquired through the same recording system as the EEG (SAGA, TMSi). Signals recorded included electromyography (EMG) activity from the right tibialis anterior and right sternocleidomastoid muscles, as well as an electrocardiography (ECG) channel with electrodes positioned bilaterally across the chest beneath each clavicle. Before electrode placement, the skin was prepped by cleaning with an alcohol wipe. Signals were sampled at 2000 Hz.

### 2.3. Band definition and filtering

Metrics were evaluated in the typical EEG frequency bands: alpha (*α*, 8–13 Hz), beta (*β*, 13–30 Hz), low gamma (*γ*_low_, 30–55 Hz), high gamma (*γ*_high_, 65–200 Hz), and broadband (1–200 Hz). Filters were implemented with second order zero-lag Butterworth filters. Band power was computed for each frequency band by integrating the power spectral density (PSD) across the band’s frequency range and normalizing by the bandwidth. The PSD was estimated using Welch’s method.

### 2.4. Preprocessing: channel selection and artifact removal

The stent-electrode device supports a maximum of 16 channels, but only 12 were connected in this participant due to incomplete insertion of the lead connectors into the IRTU header. A further 3 channels were excluded due to high impedance (>100 kΩ) or noise evident on visual inspection, resulting in 9 channels used in analyses of movement tasks. For all cued movement tasks, the vECoG recordings utilized the local referencing scheme. This approach was selected due to practical time constraints, which precluded repeating all experimental runs under multiple referencing conditions.

The scalp EEG cap supports up to 64 channels, but data were only collected for 62, as the mastoid sites (M1 and M2) were utilized for grounding. For analyses of movement tasks, the scalp EEG data were limited to central channels spanning FC3 to CP4, corresponding to the expected region of interest over the sensorimotor cortex.

To quantify the true neural signal of interest, we excluded trials contaminated with artifacts, most likely arising from myoelectric activity. The broadband data were visually inspected for trials containing large amplitude activity or transients affecting multiple electrodes simultaneously; these trials were marked as bad and excluded from further analysis (supplemental figure 1). Importantly, artifact rejection was applied jointly across modalities: if an artifact was detected in either the vECoG or scalp EEG recordings, the trial was excluded from both modalities to ensure equal trial counts and unbiased comparisons. In total, 5% of trials were excluded with 4.75% attributable to artifacts identified in the scalp EEG and 0.25% to artifacts identified in the vECoG (supplemental table 1). Due to visual inspection, artifact identification involved a degree of subjectivity; however, artifact removal likely introduced a positive bias in the scalp EEG signal quality by excluding trials with prominent EEG contamination (supplemental figure 1). The myoelectric artifacts are presumed to occur when the participant made postural adjustments, facial expressions, vocalizations, or other movements.

### 2.5. Baseline evaluation

At the beginning of the session, multiple 2-minute baseline recordings were collected under distinct experimental conditions. The participant was instructed to rest (remain still) and either stare at a fixation circle (during the eyes-open baseline) or close his eyes (during the eyes-closed baseline) until the research team indicated that the run was complete. These baseline tasks were done under two reference configurations for the stent-electrode array: using a local reference and using the IRTU reference.

Baseline activity was quantified by computing the broadband RMS amplitude for each channel over the entire duration of the baseline recording. To assess changes in neural activity between eyes-open and eyes-closed conditions, the percentage change in alpha power (8–13 Hz) was calculated relative to eyes-open condition. The percent change in alpha power was calculated for each channel and segmented brain region. For scalp EEG, brain regions were defined based on the international 10–10 system electrode nomenclature, with electrodes assigned based on the following label prefixes: frontal (Fp, AF, F, FC), central (C), parietal (P, CP), occipital (O, PO), and temporal (T, TP, FT).

### 2.6. Movement tasks

The participant performed a series of visually cued attempted movement tasks without feedback (offline). Each run consisted of 20 trials, beginning with a 5 ± 1 s rest period focused on a fixation circle, followed by a 1-s movement attempt period, where the participant was asked to attempt 1 movement. The movement conditions included both ankles, right ankle, and left ankle. Ankle movement consisted of dorsiflexion while the heel remained stationary and in contact with the ground throughout the movement. Additionally, the participant performed right ankle movement at three effort levels: regular overt movement, light effort movement, and imagery. For the light-effort condition, the participant was instructed to perform the same dorsiflexion movement but at 50% his typical effort, which was qualitatively observed to produce a reduced range of motion. For the imagery condition, the participant was asked to refrain from physical movement but to imagine the dorsiflexion movement. The participant was also instructed to perform tasks that could elicit artifactual signals, following the same trial structure. These included one run each of jaw clenching and vocalization, with the movement period consisting of a single spoken word (word list derived from [22]).

### 2.7. Quantifying movement modulation strength

To quantify the strength of neural activity from a particular movement, we measured the cross-validated Euclidean distance between two n-dimensional distributions, one derived from rest trials and the other from movement trials. Cross-validated Euclidean distance was used to quantify differences in neural activity for a particular movement, as it incorporates information across multiple channels while reducing bias in the presence of noise [23]. Preprocessing consisted of bandpass filtering, extracting the envelope (via Hilbert transform), and smoothing with a 250 ms window. The RMS was computed for each trial and *z*-scored to the mean and standard deviation of the rest trials within the corresponding run. The distance between two multivariate distributions (e.g. a distribution of RMS amplitude vectors corresponding to the rest condition [distribution 1] and a distribution of RMS amplitude vectors corresponding to the movement (i.e. right ankle movement) [distribution 2]) was quantified using cross-validated Euclidean distance as described in Willet *et al* [23]. These distributions are *n*-dimensional, where *n* is the number of channels. The cued movement runs initially contained an equal number of trials in each class (rest and movement); however, class sizes could become unequal following the removal of trials containing artifacts. When class sizes were equal, leave-one-trial-out cross-validation was used. For unequal class sizes, the data were partitioned into the number of folds equal to the number of trials in the smallest class. Trials were treated as the observational unit for statistical inference. To account for the uneven number of channels between modalities, the cross-validated Euclidean distance was normalized to the square root of the number of channels.

95% confidence intervals were estimated using jackknife resampling, as prior work has shown that bootstrap resampling can introduce an upward bias in the cross-validated distance estimates by resampling identical data points [23]. Significant separation between the rest and movement conditions was assessed by determining whether the 95% jackknife confidence interval for the cross-validated Euclidean distance excluded zero.

Differences in neural modulation strength between vECoG and scalp EEG were assessed using a paired delete-one jackknife. A paired delete-one jackknife was performed by omitting the same trial from both recording modalities for each jackknife replication. The paired difference between modalities was computed for each leave-one-out estimate, and jackknife pseudovalues were constructed from the full-sample and leave-one out estimates. The pseudovalues were used to estimate the standard error of the full-sample modality difference and statistical significance was assessed using a Wald test with a normal approximation.

To account for multiple comparisons across frequency bands, p-values were adjusted using the Benjamini–Hochberg false discovery rate (FDR) procedure [24]. Throughout the manuscript, statistical significance follows typical denotation: * *p* ⩽ 0.05, ** *p* ⩽ 0.01, *** *p* ⩽0.001, and **** *p* ⩽ 0.0001. Given the single-participant study design, comparisons are intended to characterize within-participant differences and should be treated as exploratory rather than population-level inference.

### 2.8. Quantifying graded movement effort

To assess the capacity of the two modalities to differentiate graded motor exertion levels, three effort levels were analyzed: regular overt movement, light effort movement, and motor imagery. To compare the neural modulation between the three exertion levels, cross-validated Euclidean distance was utilized (described earlier). Pairwise differences in modulation strength between effort levels were assessed using restricted permutation tests. Effort labels were permuted while preserving the movement/rest labels. The cross-validated Euclidean distance was recomputed for each permutation, and two-sided *p*-values were obtained from the resulting null distribution. *P*-values were adjusted using the Benjamini–Hochberg FDR procedure to account for multiple comparisons across frequency bands.

To confirm that the participant was grading the output as requested, we utilized the EMG channel placed on the right tibialis anterior muscle, which was recorded simultaneously during the runs. The EMG activity was bandpass filtered between 30 and 400 Hz, rectified using the analytic envelope (Hilbert transform), and smoothed with a 10 Hz lowpass filter. The mean EMG envelope was calculated for each movement trial. To evaluate if there were significant differences between mean effort levels, an independent samples *t*-test was utilized.

### 2.9. Quantifying lateralized movements

We assessed if left- and right-sided movements exhibit topographical differences across the two modalities by quantifying left ankle, right ankle, and both ankle movement. Two runs were collected for each movement type and subsequently combined, resulting in 40 repetitions per movement. The strength of neural modulation was quantified between rest and movement trials per channel using cross-validated Euclidean distance and plotted on electrode activation maps for each modality. Additionally, modulation-weighted centroids were computed from the channel distances and overlaid on the 2D maps. During centroid estimation, the stent-electrode array was modeled with an assumed diameter of 8 mm; however, this is expected to vary slightly based on participant anatomical differences. To quantify uncertainty in the centroid location, trial-level bootstrap resampling was performed, with centroids recomputed for each resample (1000 total) to generate a sampling distribution. Bootstrap resampling was used as a standard nonparametric approach to estimating uncertainty from the underlying trial distribution. A 95% confidence interval ellipse was computed from the covariance of bootstrap centroid estimate.

The vECoG electrode activation map was displayed in the coronal plane because the array’s geometry and cortical location suggest the left–right differences in neural activation would be strongest in this orientation. Varying views of the stent-electrode array are shown in supplemental figure 2. Importantly, due to limitations of the current stent-electrode array design, the array’s orientation in the coronal plane is unknown and is likely rotated relative to the orientation shown. Furthermore, individual electrodes cannot be definitively assigned to the left or right hemisphere.

To confirm lateralized cortical activation, pre-implant functional neuroimaging was analyzed during left and right ankle movements (neuroimaging analysis described below).

### 2.10. Artifact quantification

Prompted artifacts, including jaw clenching and vocalization, were quantified using a baseline-to-artifact ratio, which was defined as the ratio of root mean square (RMS) of the ongoing brain activity to the RMS during the cued artifact period. For both the artifact and ongoing trials, RMS was calculated for each trial and then averaged across trials.

\begin{equation*}\mathrm{Baseline} - \mathrm{to} - \mathrm{artifact}{\text{ }}\mathrm{ratio} = \frac{{{\mathrm{RMS}_{\mu ,{\text{ }}\mathrm{ongoing}}}}}{{{\mathrm{RMS}_{\mu ,{\text{ }}\mathrm{artifact}}}}}\end{equation*}

A ratio of 1 indicates that the artifact is indistinguishable from ongoing activity, while 0 would be an artifact approaching infinity. Artifact ratios were calculated using broadband data (1–200 Hz).

Spontaneous and environmental artifacts were identified in the baseline data, including eye blinks, residual cardiac activity, and 60 Hz line noise. Eye blinks were identified using peak detection on the Fp1 channel (SciPy library [25]). For each blink, a 500 ms segment (±250 ms) was extracted from all channels. To quantify the relative blink amplitude, peak-to-peak amplitude was computed from the average blink waveform. To normalize against background activity, amplitudes were *z*-scored to the mean and standard deviation of the channel’s activity outside of the blink segments. Additionally, eye blinks were also quantified using the baseline-to-artifact ratio described above using the 500 ms window as the artifact window.

To identify electrocardiac contamination, the neural recordings were assessed by comparing against the ECG lead recorded cutaneously. The peaks of the R-waves in the ECG were identified using peak detection, and ECG segments were extracted around each peak. ECG strength in the neural data was quantified by projecting the neural data segments time-locked to the R-peak onto the average ECG waveform template. The normalized ECG contamination for each channel was defined as the RMS projection of the neural data onto the ECG template, normalized by the RMS projection of the ECG itself:

\begin{equation*} {\mathrm{ECG}_{\mathrm{strength}{\text{ }}}} = \sqrt {\frac{1}{K}\mathop \sum \limits_{k = 1}^K {{\left( {\frac{{{y^k} \cdot t}}{{{{\left| {\left| t \right|} \right|}^2}}}} \right)}^2}} \end{equation*}

where ${y^k}$ is the *k*th ECG epoch and *t* is the ECG template.

A score of 0 indicates no ECG-related structure in the neural data, while a score of 1 indicates the signal is as strong as the external ECG itself.

60 Hz noise was quantified by measuring the prominence of the 60 Hz peak in the PSD. For each channel, prominence was calculated as the difference between the peak power within the 59–61 Hz range and the average power across the surrounding frequency bands (50–58 Hz and 62–70 Hz).

Artifact metrics (e.g. baseline-to-artifact or peak prominence) are reported as mean ± standard deviation unless otherwise noted. For comparisons with a reference value (e.g. 1 for the baseline-to-artifact ratio and 0 for peak prominence), a two-sided one-sample permutation test was performed using channel-level observations as the permutation unit. The null distribution was formed by randomly sign-flipping channel-level deviations from the reference value, corresponding to the null hypothesis of no systematic difference from the reference value.

Differences between scalp EEG and vECoG were evaluated using two-sided independent-samples permutation tests at the channel level. Channel observations from both modalities were pooled, modality labels were randomly reassigned, and the difference in mean artifact metric between vECoG and scalp EEG was recalculated for each permutation. This generated the null distribution expected if channel-level observations were exchangeable between modalities. All permutation tests used 10 000 resamples. For artifact quantification, because data were obtained from a single-participant and channel-level observations are likely spatially correlated and not independent, statistical comparisons were treated as exploratory, within-subject analyses and do not support population-level inference. Effect sizes were qualified as the mean difference between modalities (Δ), and standardized effect sizes were quantified using Cohen’s *d* based on the pooled standard deviation. No corrections for multiple comparisons were made as only the broadband frequency band was evaluated.

### 2.11. Functional neuroimaging data collection and analysis

Prior to implantation, the participant underwent functional MRI scans while performing left or right ankle movements to measure the ability to functionally activate cortical regions during movement. The participant was instructed to repeatedly flex and extend their ankle, guided by audio- (i.e. Avotec MR-compatible headphones) and text-cues projected into the scanner bore. A block design was used, consisting of 15 s OFF (‘rest’) period followed by a 15 s ON (‘move’) period, repeated 8 times, followed by a final 15 s rest period. There was 1 run testing the left ankle and 1 run testing the right ankle. MRI images were obtained using a 3-Tesla scanner (Siemens Magnetom Biograph mMR) and a 32-channel head coil. The parameters of the structural and functional scans have been previously reported in detail (participant 10 in Schone *et al* [18]).

Functional MRI data processing follows the methods described in Schone *et al* [18]. This included reconstructing the cortical surface, preprocessing, and time-series statistical analysis to determine the significance of functional activity based on the blood oxygenation level-dependent (BOLD) signal registered to the participant’s structural image. Two regions of interest were defined based on the participant’s cortical surface: medial motor cortex and somatosensory cortex.

To visualize ankle-related neuromotor activity, we minimally thresholded each *z*-stat to 4.5 (left ankle shown in red and right ankle shown in blue) and displayed the activity on representative slices. Finally, to quantify whether there is lateralized hemispheric activity in the two regions when performing the movements, we extracted the average *z*-stat within each voxel during left and right ankle movements. The voxel-wise activity in each hemispheric region for each movement was quantified. Independent samples *t*-test was used to compare activity between the hemispheres.

## 3. Results

**Figure 1.**
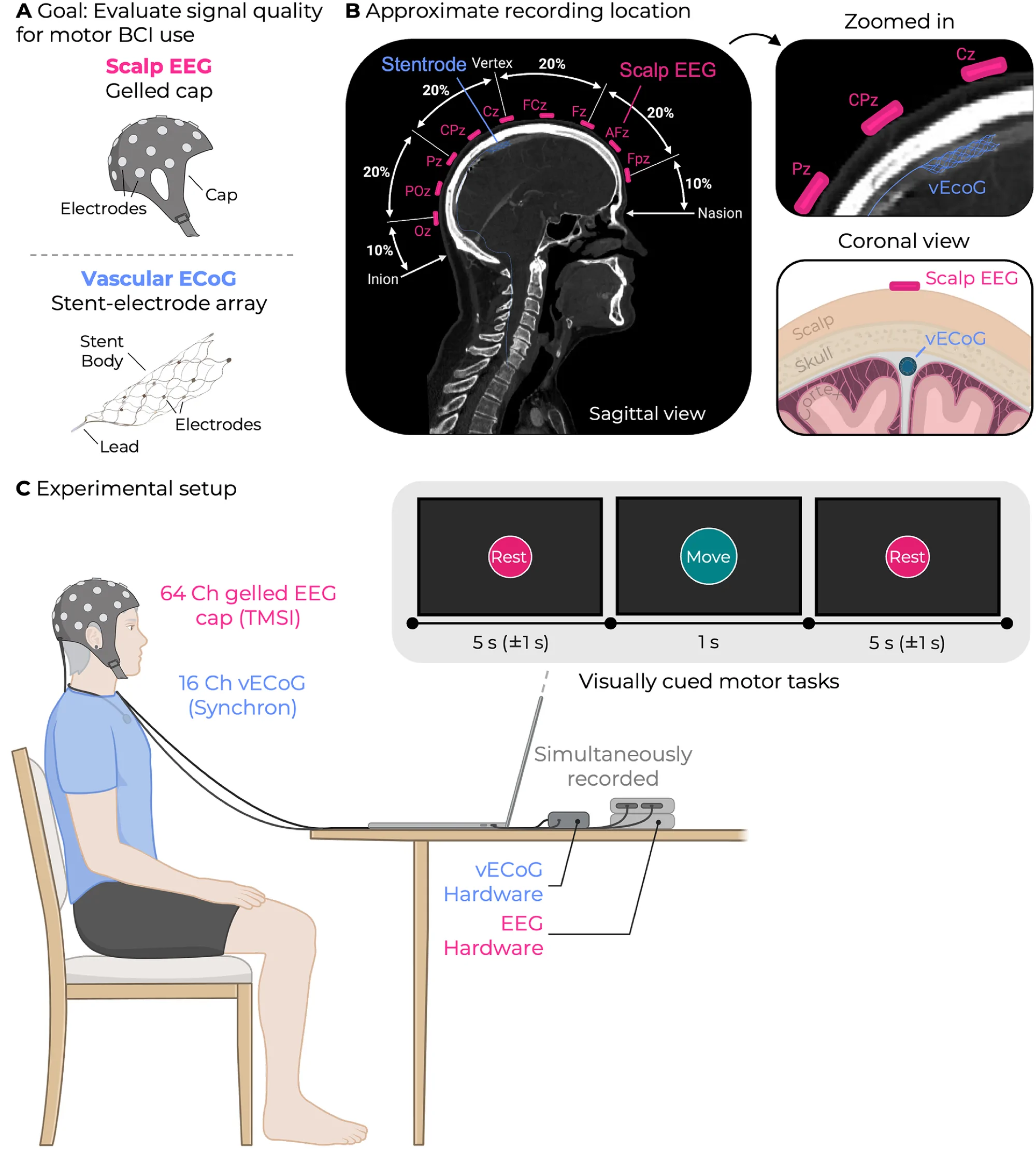
Experimental setup for simultaneous scalp EEG and vECoG recordings. (A) We evaluated signal quality relevant to motor BCI use by directly comparing two recording modalities: a non-invasive scalp EEG cap and an implanted stent-electrode array. (B) A midline sagittal slice of the 3-month post-implant CT showing the approximate location of the stent-electrode array (blue array overlaid on CT). The location of the midline scalp EEG electrodes (pink) are approximated based on 10% spacing from the inion to nasion. (C) A zoomed-in version of the CT in panel B showing the stent-electrode array closer up. (D) A sketch of the approximated device position in the coronal plane. (E) Schematic of the recording setup. Neural activity was simultaneously recorded from a 64-channel gelled EEG cap and a 16-channel stent-electrode array. The participant followed visual cues on a laptop positioned in front of him. Each trial consisted of a 5 ± 1 s rest period followed by a typical 1-s movement attempt period, with 20 trials presented per run. Parts of this figure were created with BioRender.com. Abbreviations: EEG, electroencephalography; vECoG, vascular electrocorticography.

To characterize the relative advantages and limitations of signal quality in vECoG and scalp EEG recordings, neural signals were recorded simultaneously from both modalities in one participant with severe upper limb paralysis due to ALS. EEG was recorded using a 64-channel cap with gelled electrodes, and vECoG from a 16-channel stent-electrode array implanted in the SSS. Figure 1 shows the approximate locations of the recording electrodes based on the post-implant CT and the experimental setup.

To investigate signal quality features of the scalp EEG and vECoG, we separately quantified both signal and noise characteristics during cued motor tasks and baseline resting activity. Signal-related metrics include strength of movement-related modulation, graded amplitude control, and the degree of spatial lateralization. Noise-related metrics include both spontaneous artifacts, such as 60 Hz line noise, ECG contamination, and eye blink artifacts, and prompted artifacts induced by jaw clenching and vocalization.

### 3.1. Attempted movement modulates neural signals

**Figure 2.**
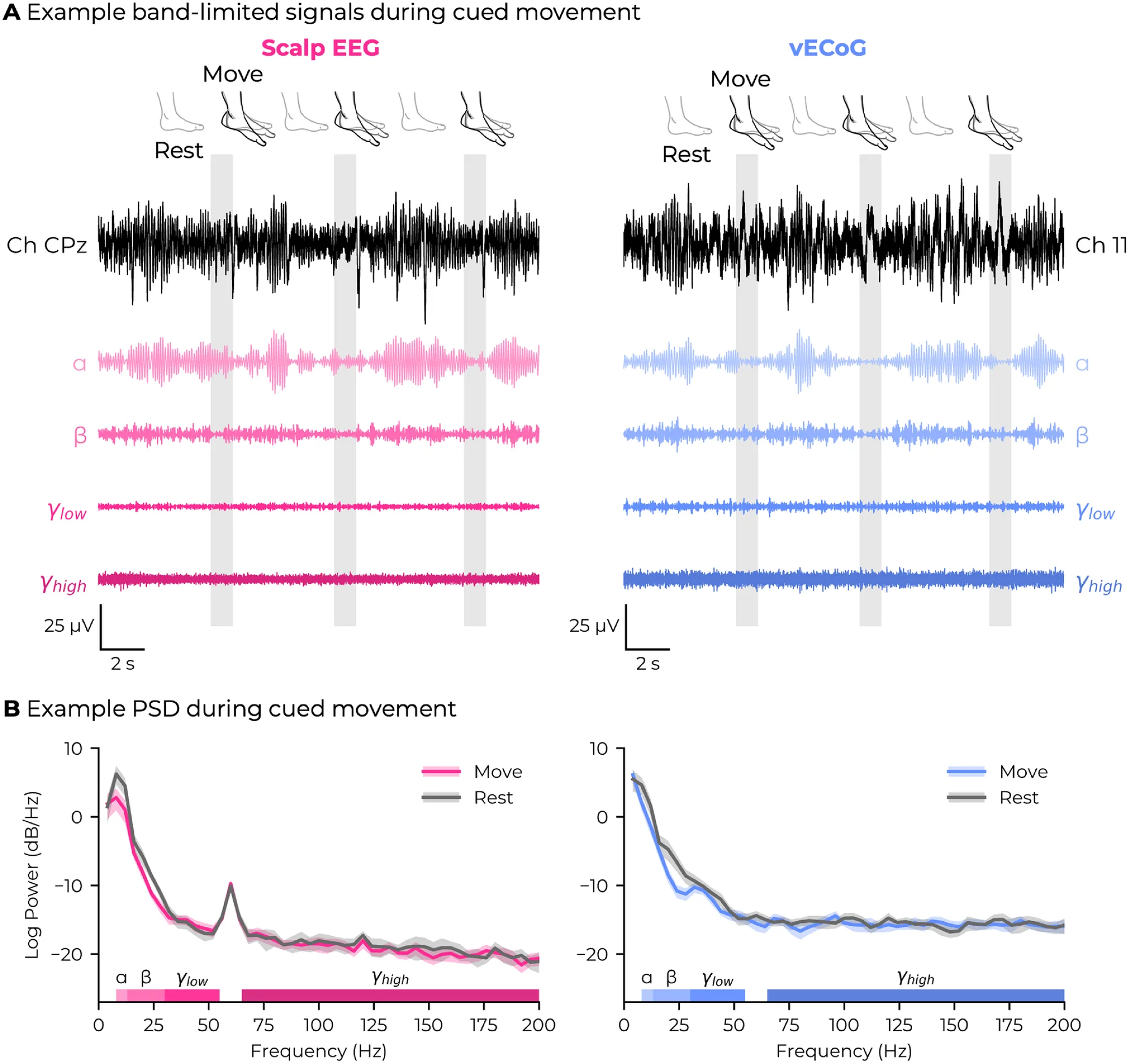
Example neural activity during attempted movement recorded simultaneously from scalp EEG and vECoG. (A) Broadband (1–200 Hz) and band limited data in alpha (*α*, 8–13 Hz), beta (*β*, 13–30 Hz), low gamma (*γ*_low_, 30–55 Hz), and high gamma (*γ*_high_, 65–200 Hz) from a single channel during movement of the left ankle. The shaded gray regions indicate the visual cues to move. Scalp EEG (pink, left) and vECoG (blue, right) are shown side by side for comparison. (B) Power spectral density (PSD) during movement (color) and rest (gray) over the entire run. Solid lines show the mean PSD across 20 trials; shaded areas indicate the 95% confidence interval (CI) of the mean. The color bars at the bottom show the frequency band definitions used.

A representative example of simultaneously recorded scalp EEG and vECoG signals during left ankle movement is shown in figure 2. In both modalities, movement-related de-synchronization is evident in the lower-frequency bands, particularly in the alpha and beta bands.

**Figure 3.**
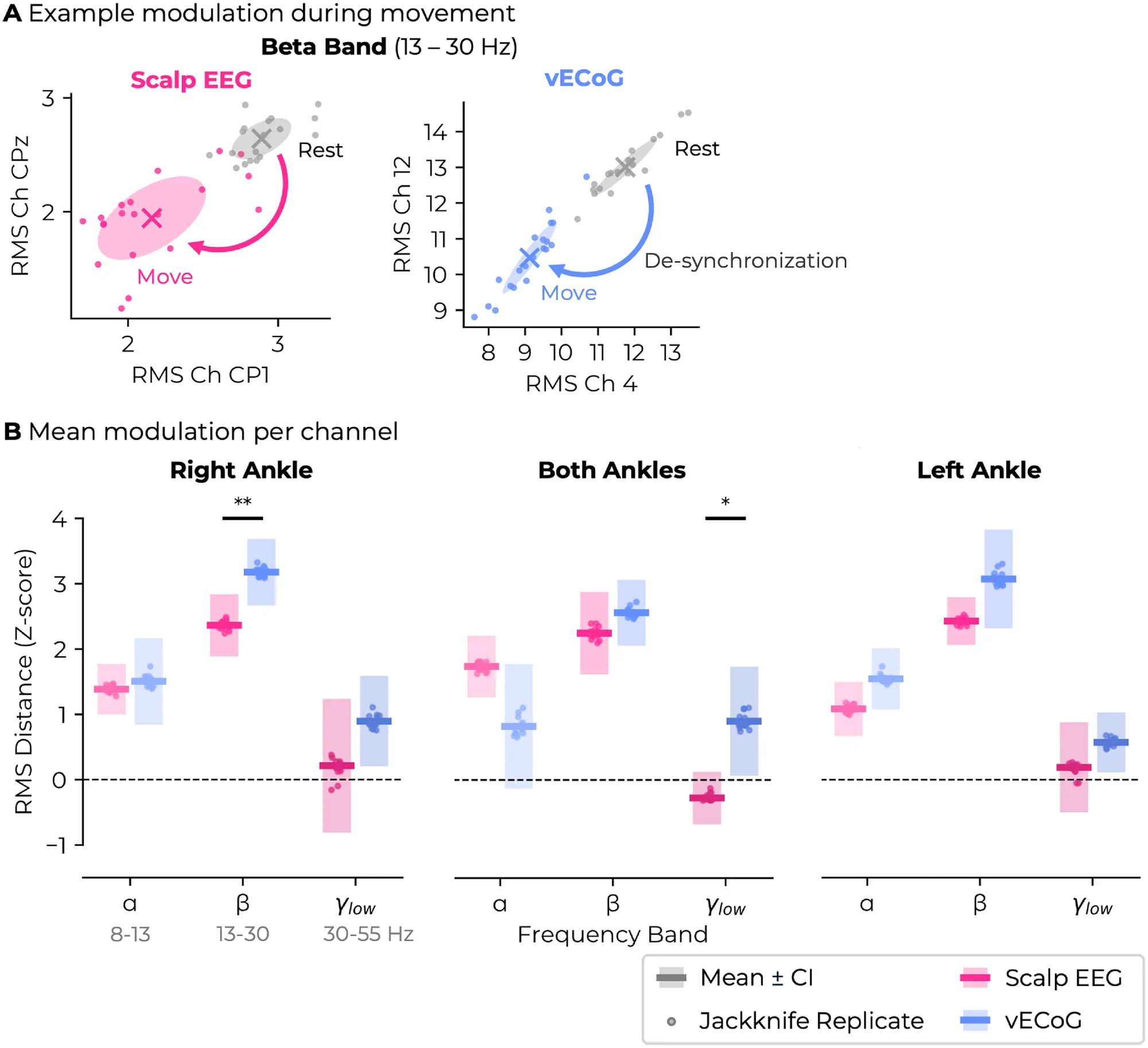
Strength of movement-related modulation. (A) Example 2D visualization of the modulation between move and rest in the two highest modulated channels for each modality. Each scatter point is the beta band-limited RMS from a single rest (gray) or move attempt (colored). Shaded ellipse shows the covariance and X marks the centroid. The cross-validated Euclidean distance is computed as the distance between the rest and move condition vectors formed in an *N*-dimensional space, where *N* are the number of channels utilized for each modality. RMS, root-mean squared. (B) Mean per channel signal modulation from rest as measured with cross-validated Euclidean distance for scalp EEG (pink, left bars) and vECoG (blue, right bars) across frequency bands: alpha (*α*, 8–13 Hz), beta (*β*, 13–30 Hz), low gamma (*γ*_low_, 30–55 Hz). The thick line shows the mean and shading denotes 95% confidence interval (CI) of the mean across 20 repetitions of the movement. Each scatter point shows one jackknife replicate. The dashed black line at 0 represents no change from ongoing (baseline, resting) activity. Significant differences in modulation between modalities were assessed using an exploratory, within-subjects Wald test. Asterisks denote significance level: *p ⩽ 0.05, **p ⩽ 0.01, ***p ⩽ 0.001, and ****p ⩽ 0.0001.

We first compared the quality of neural signals recorded during cued movements between the two modalities. Figure 3 quantifies the mean modulation per channel during attempted movement. Across both modalities, significant modulation relative to rest was observed in the alpha and beta bands, and in some cases the low gamma band for vECoG, as evidenced by the 95% confidence intervals not including zero. For the majority of frequency bands and movements, mean modulation was greater in vECoG than the scalp EEG, with several comparisons reaching statistical significance (within-subjects Wald test, *p* < 0.05). An example of channel-wise motor modulation is shown in supplemental figure 3.

### 3.2. Neural motor signal modulation scales with effort

**Figure 4.**
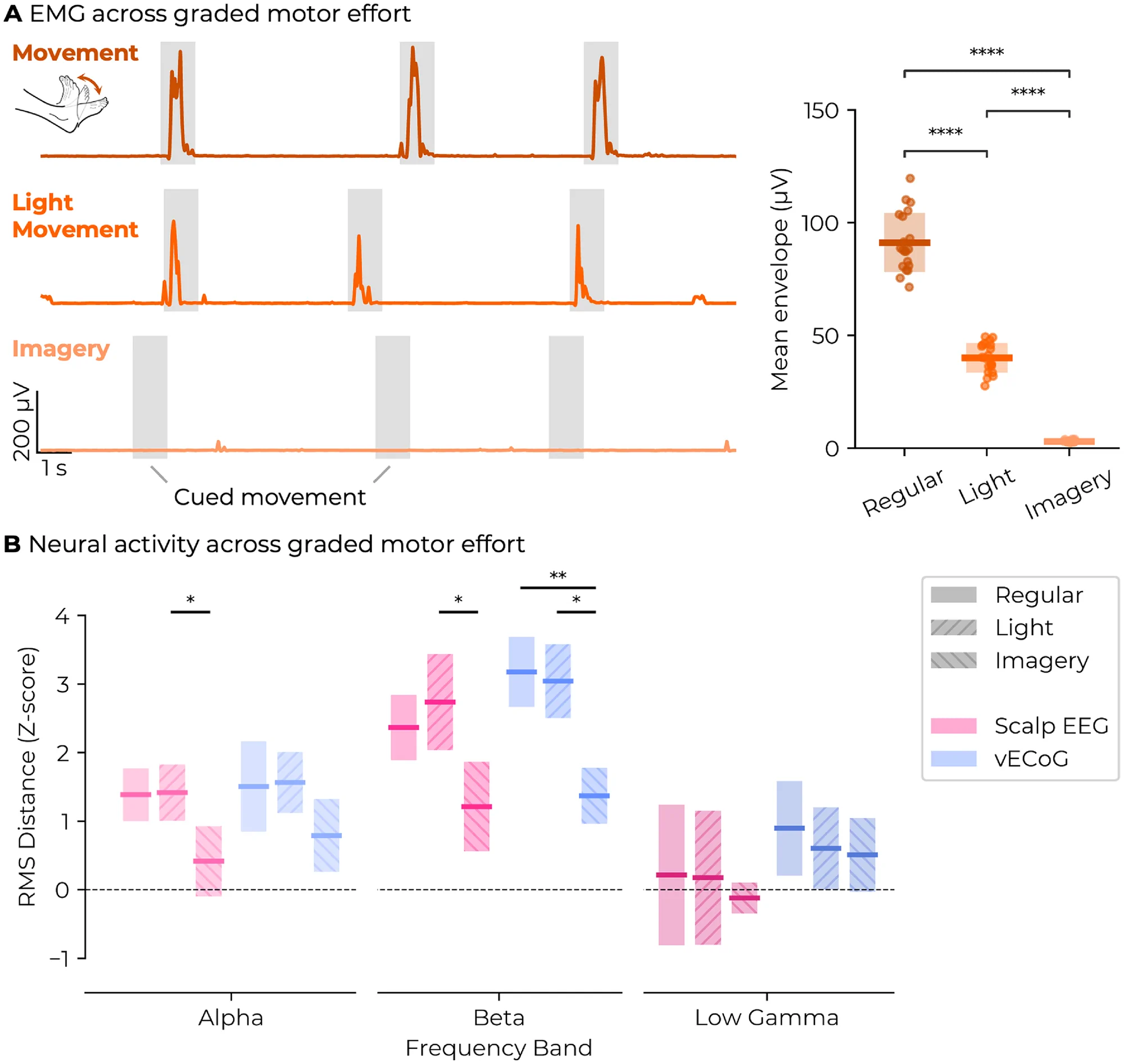
Neural motor signal activity across graded exertion. (A) *Left:* Smoothed electromyography (EMG) envelopes recorded from the right tibialis anterior demonstrates the participant is grading output as requested. Each trace corresponds to a single run from right overt ankle movement (top), light effort movement (middle), and motor imagery (bottom). The gray shaded region shows the visual cue to move. *Right:* Mean EMG envelope amplitude during the cued movement periods. Each scatter point is a single trial (20 trials of each movement). The thick line shows the mean and shading denotes 1 standard deviation. All pairwise comparisons were significantly different (within-participant *t*-test, *p* < 0.001). RMS, root-mean squared. (B) Mean per channel modulation from rest as measured with cross-validated Euclidean distance for scalp EEG (pink) and vECoG (blue) across the three effort levels of right ankle movement. The thick line shows the mean and shading denotes 95% confidence interval of the mean across 20 repetitions of the movement. The dashed black line at 0 represents no change from ongoing (baseline, resting) activity. Significant differences in modulation between effort levels were assessed using within-participant permutation tests with FDR correction for multiple frequency bands. Asterisks denote significance level: *p ⩽ 0.05, **p ⩽ 0.01, ***p ⩽ 0.001, and ****p ⩽ 0.0001.

Next, we investigated whether neural motor signals were modulated by movement effort levels, specifically across regular overt movement, light (low effort) movement, and motor imagery. To confirm that the participant was actually grading the output as requested, we compared the muscle activity recorded from the tibialis anterior across the three effort levels (figure 4(A)). Regular overt movement, light movement, and imagined movement had a mean EMG envelope of 91.3, 40.1, and 3.2 *μ*V, respectively. Pairwise comparisons show that each condition was significantly different from the others (independent samples sample *t*-test, *p* < 0.001). The EMG power during the *light* and *imagery* conditions correspond to an average of 43.9% and 3.5% of the *regular* effort movement amplitude, respectively.

Given that the EMG data demonstrated a strong difference in amplitude with movement effort, we next examined whether neural signals from either modality exhibited modulation that correlated with the effort level. Figure 4(B) quantifies the mean neural modulation per channel during regular overt, light, and imagined right ankle movement with the beta band showing some statistical significance. In the beta band, vECoG modulation during *light* movement averaged 95.8% of *regular* movement, while *imagery* had a considerably reduced modulation at 43.1% of *regular* movement. In contrast, scalp EEG exhibited a slight increase during light movement at 111.5% of *regular* movement and had a similar reduction in modulation during *imagery*, at 51.2% of *regular* movement.

### 3.3. Comparing spatial localization of lateralized movements

**Figure 5.**
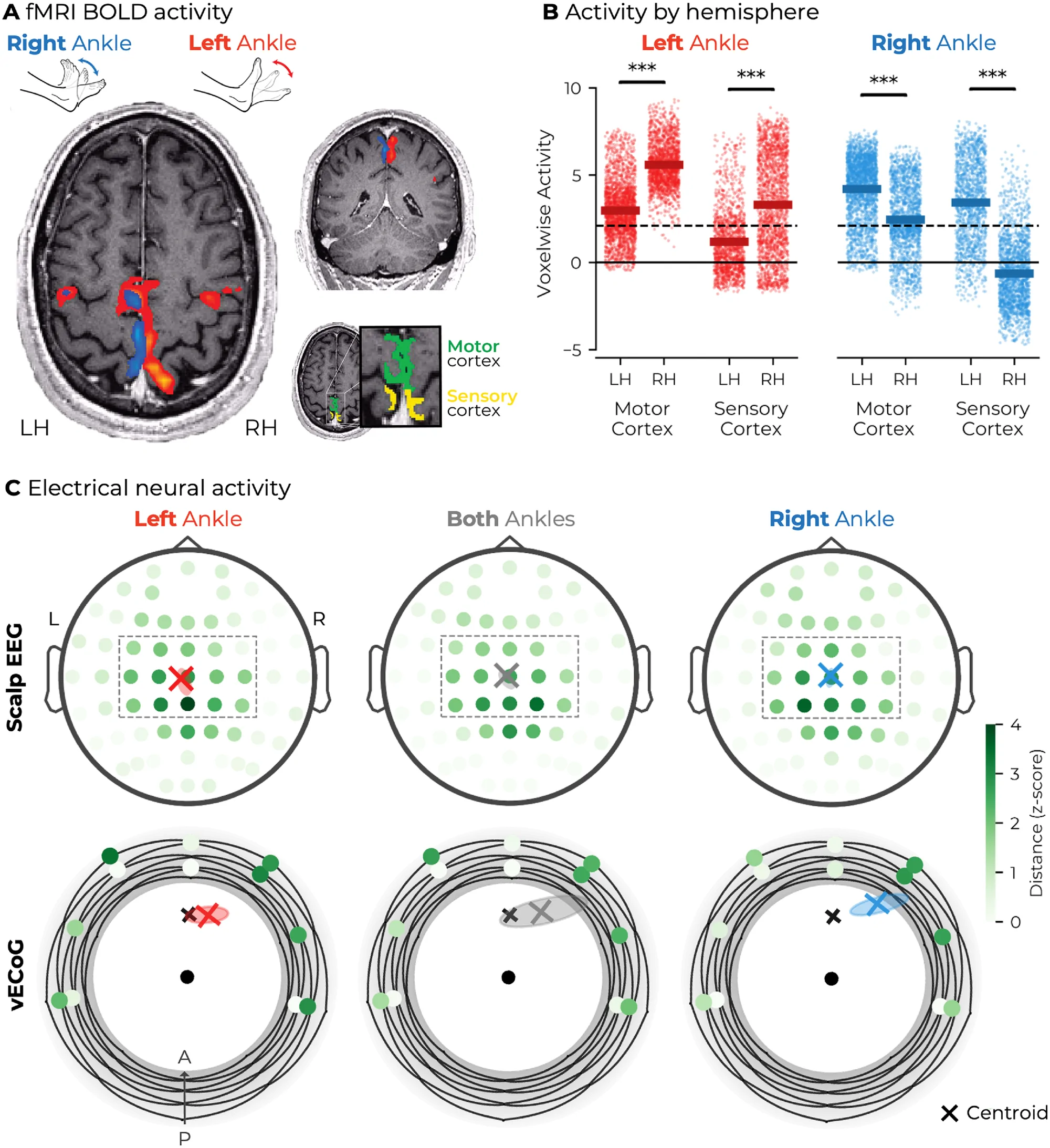
Comparing lateralized activity. (A) Right (blue) and left (red) ankle cortical activation (fMRI BOLD signal) from some representative slices during cued movement. The defined regions of interest for medial motor cortex (green) and (somato)sensory cortex (yellow). fMRI, functional magnetic resonance imaging; BOLD, blood oxygenation level-dependent. (B) The lateralized hemispheric activity when performing the movements in the two regions of interest. Each scatter point is the average *z*-stat voxel-wise activity during left or right ankle movements compared to rest. The thick line shows the mean activity. Left and right hemisphere activity was significantly different in each region of interest (within-participant comparison, independent samples *t*-test, *p* < 0.001). The dashed line shows *z*-statistic = 2.1, which is the threshold for activity statistically different from rest at *p* < 0.05 (uncorrected, two-tailed). LH, left hemisphere; RH, right hemisphere. Asterisks denote significance level: *p ⩽ 0.05, **p ⩽ 0.01, ***p ⩽ 0.001, and ****p ⩽ 0.0001. (C) Electrode activation maps for scalp EEG (top row) and vECoG (bottom row). Each electrode shows the mean channel-wise activity during the specified ankle movement compared to rest (measured within the beta band with cross-validated Euclidean distance, *z*-scored) across 40 repetitions of the movement. Zero represents no change from ongoing (baseline, resting) activity. Scalp EEG electrode layout is displayed from a superior view. vECoG electrode layout is displayed in the coronal plane, viewed along the central axis of the stent-electrode from a posterior perspective. The large X marks the weighted centroid and ellipses the confidence interval of the centroid. The centroid for scalp EEG is calculated using the channels around the sensorimotor strip (dashed box). The smaller, black x in the vECoG layout marks the unweighted centroid. Annotations on electrode layout: L, left; R, right; P, posterior; A, anterior.

Given the midline placement of the stent-electrode array, this may enable advantages in localization of sources from the left and right hemispheres. Therefore, we next investigated whether lateralized activity patterns were observed during unilateral movements. First, we wanted to confirm the participant could generate lateralized hemispheric activity during unilateral movement. To this end, we quantified the pre-implant fMRI BOLD responses to cued left and right ankle movements. Activity from representative MRI slices is shown in figure 5(A). The voxel-wise activity in each hemispheric region for each movement is displayed in figure 5(A). As expected, the activation was the strongest in the contralateral motor cortex. Left and right hemisphere activity was significantly different for each movement in each region of interest (paired *t*-test, *p* < 0.001). Of note, this activity is measured at a voxel size 2mm^3^ which provides a much higher density spatial sampling than either array measures.

Considering the fMRI data revealed a highly lateralized response for ankle movements, we next examined whether this laterality was similarly observed in the vECoG or scalp EEG data. Figure 5(B) quantifies the per channel neural modulation comparing right, left, and both ankle movement. In the beta band, scalp EEG electrodes exhibited a modest modulation across the electrodes over the sensorimotor strip, with the centroid of activity localized near the midline, consistent with established somatotopic representations of the lower-limb [26–28]. vECoG demonstrated more variability in channel-wise modulation across the left and right movement, with elevated modulation concentrated on a subset of electrodes. Centroid shifts between left and right ankle movement did not differ significantly for either modality, as indicated by overlapping confidence intervals.

### 3.4. Prompted artifacts

**Figure 6.**
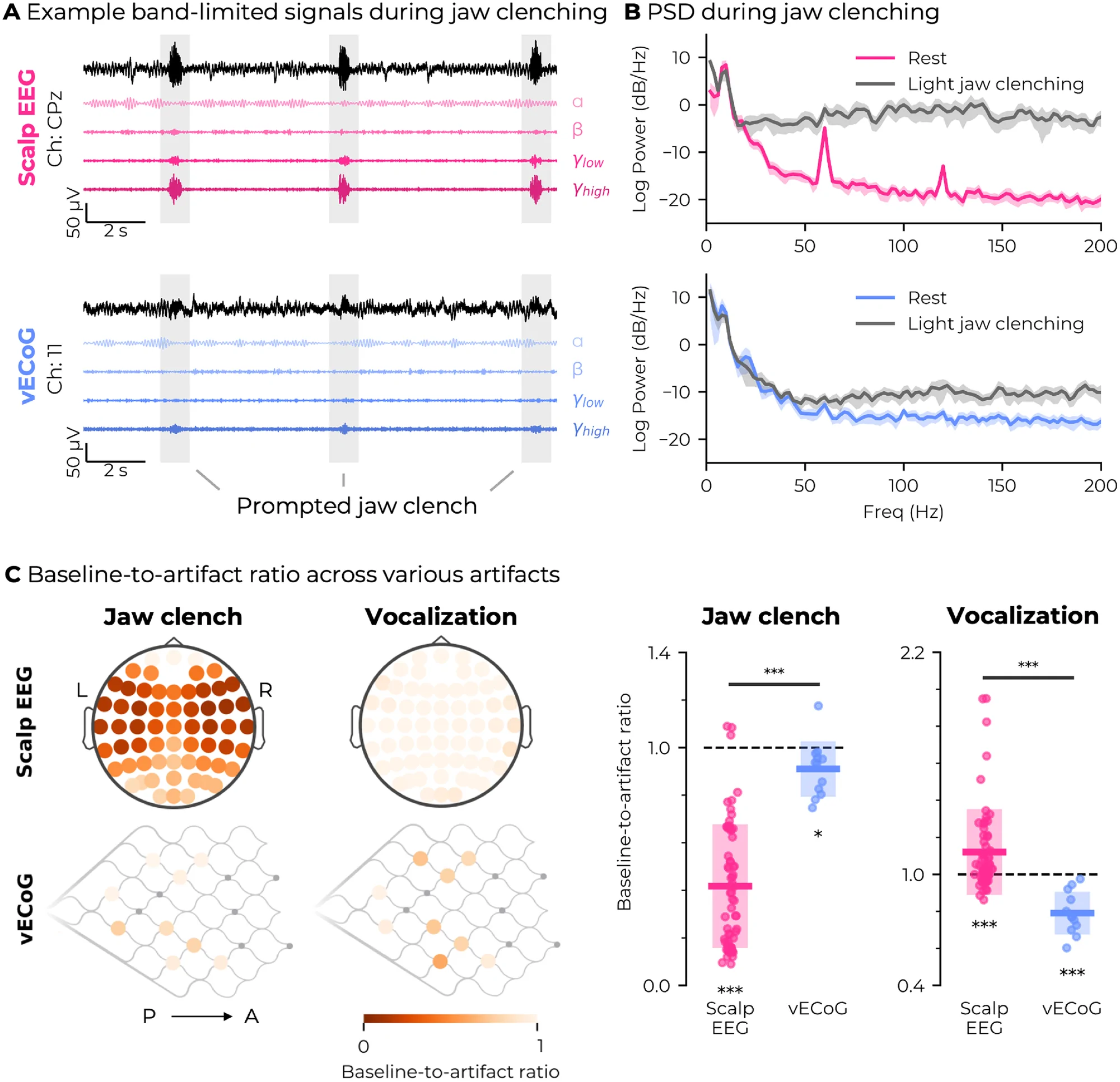
Simultaneously recorded prompted artifacts from scalp EEG and vECoG. (A) Example channel broadband and band-limited signals during visually prompted jaw clenching (shaded gray box). Frequency bands show: broadband (black, 1–200 Hz) and band limited data in alpha (*α*, 8–13 Hz), beta (*β*, 13–30 Hz), low gamma (*γ*_low_, 30–55 Hz), and high gamma (*γ*_high_, 65–200 Hz) from a single channel. (B) Mean power spectral densities (PSD) during rest (colored) and jaw clenching (gray), with shading indicating 95% confidence interval of the mean. (C) Baseline-to-artifact ratios for each channel shown on the device layout and summarized across channels. The thick line shows the mean and shading denotes 1 standard deviation. The dashed black line at 1 represents no change from ongoing (baseline, resting) activity. The stars below indicate if the mean ratio was significantly different from 1 (exploratory, within-participant permutation tests). Significant differences between modalities were assessed using exploratory, within-participant permutation tests. Asterisks denote significance level: *p ⩽ 0.05, **p ⩽ 0.01, ***p ⩽ 0.001, and ****p ⩽ 0.0001. Annotations on electrode layout: *L*, left; *R*, right; *P*, posterior; *A*, anterior.

Non-neural artifacts would be expected to differentially affect invasive and non-invasive neural recordings, motivating our next evaluation of the susceptibility of vECoG and scalp EEG to common artifacts. The participant was asked to perform actions anticipated to induce artifacts, including jaw clenching and vocalization, to characterize their relative impact on each recording modality. Example time series and PSD during these tasks is shown in figure 6(A). To quantify the effects across the array, the baseline-to-artifact ratio was computed.

We first examined the effects of jaw clenching, a well-established source of EMG contamination in the higher-frequency bands [29, 30]. As expected, the jaw-clenching most strongly affected electrodes around the temporalis muscle, specifically the lateral frontal, frontal-temporal, and temporal channels on the scalp EEG (figure 6(B)). On average, the baseline-to-artifact ratio for jaw clenching was 0.42 ± 0.26 for scalp EEG (permutation *p*-value = 0.0002) and 0.91 ± 0.12 for vECoG recordings (permutation *p*-value = 0.000488). Indeed, scalp EEG was significantly lower (Δ = 0.49, Cohen’s *d* = 2.02, permutation *p*-value = 0.0002), indicating greater artifact contamination in the EEG than in vECoG during jaw clenching. An example of the baseline-to-artifact ratio separated by frequency band is shown in supplemental figure 4.

Given the involvement of facial and jaw musculature during speech, we next examined artifacts due to vocalization. On average, the baseline-to-artifact ratio for vocalization was 1.12 ± 0.23 for scalp EEG (permutation *p*-value 0.0002) and 0.79 ± 0.12 for vECoG recordings (permutation *p*-value 0.00049). The baseline-to-artifact ratio above 1 indicates de-synchronization during the vocalization period; this modest de-synchronization in the lower-frequency bands likely reflects neural activity related to speech production. The vECoG baseline-to-artifact ratio was significantly lower than the scalp EEG (Δ = − 0.33, Cohen’s *d* = − 1.51, permutation *p*-value 0.0002).

While it is expected that these movements produce artifactual signals, it should be noted that these signals cannot be definitively attributed to artifacts alone. The observed changes may reflect true cortical activity, or a combination of cortical activity and artifact sources, rather than pure extra-cortical sources. However, the strong concurrent activation between the neural recording (especially temporal) and muscle activity suggests that muscle activity is the dominant source (supplemental figure 5).

### 3.5. Spontaneous artifacts

To further assess how common physiological and environmental artifacts differentially impact each modality, we next analyzed resting-state, eyes-open recordings to characterize the relative impact of blink, ECG, and line noise artifacts.

**Figure 7.**
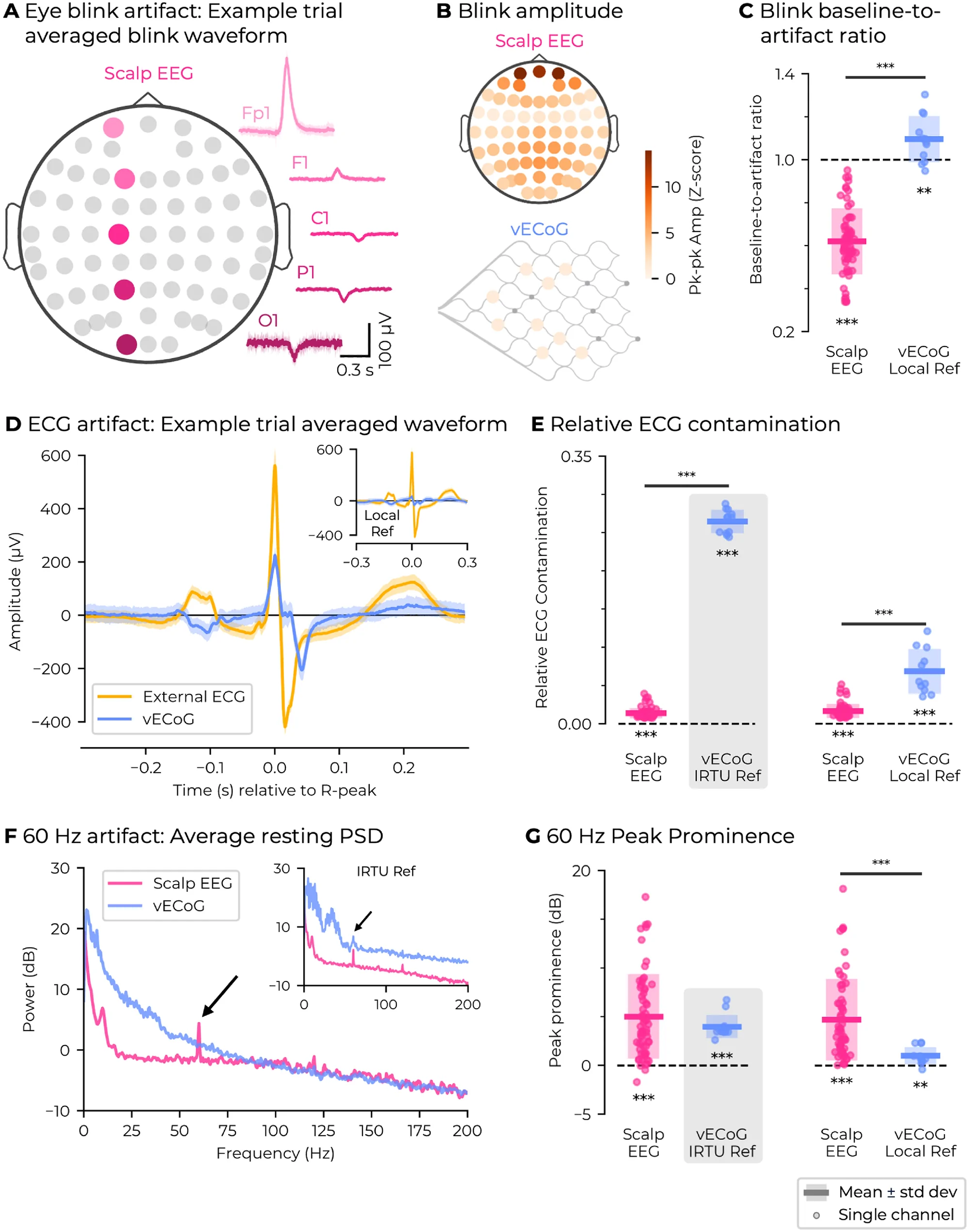
Simultaneously recorded spontaneous artifacts from scalp EEG and vECoG. Data were obtained from the eyes-open resting-state baseline recordings. (A) Average blink waveform across five representative scalp EEG channels. (B) Relative blink amplitude by channel, quantified as the peak-to-peak amplitude within the blink window and *z*-scored against non-blink segments. (C) Baseline-to-artifact ratio for the blink artifacts across all channels. The thick line shows the mean and shading denotes 1 standard deviation. The dashed black line at 1 represents no change from ongoing (baseline, blink-free) activity. The stars below each bar indicate if the mean ratio differed from 1 (exploratory, within-subject one-sample permutation test). The stars above indicate whether differences between modalities were significant (exploratory, within-subject permutation test). (D) Mean external ECG waveform (yellow) and residual ECG recorded with the vECoG (referenced to IRTU) (blue), time-locked to the R-peaks. Shading denotes ±1 standard deviation. The small inset in the upper right shows the waveform from vECoG reference locally to an electrode on the stent. (E) Relative ECG contamination, quantified as the similarity between the mean external ECG waveform and waveforms from each channel of the vECoG and scalp EEG. Black dashed lines indicate 0 or no ECG contamination. The stars below each bar indicate if the mean ECG contamination differed from 0 (exploratory, within-subject one-sample permutation test). The stars above indicate whether differences between modalities were significant (exploratory, within-subject permutation test). (F) Average power spectral densities (PSD) during rest for the scalp EEG (pink) and vECoG (blue) with local reference. The small inset in the upper right shows the PSD from the vECoG IRTU reference. (G) The 60 Hz peak prominence, defined as the difference between the peak power in the 59–61 Hz range and the average power in the surrounding bands (50–58 Hz and 62–70 Hz). Black dashed lines indicate 0 or no discernible 60 Hz peak. The stars below each bar indicate if the mean 60 Hz contamination differed from zero (exploratory, within-subject one-sample permutation test). The stars above indicate whether differences between modalities were significant (exploratory, within-subject permutation test). Asterisks denote significance level: *p ⩽ 0.05, **p ⩽ 0.01, ***p ⩽ 0.001, and ****p ⩽ 0.0001.

First, we examine blink artifacts, which were easily identified in the scalp EEG by the large transients in channels Fp1 and Fp2. Examples of the stereotyped, time-locked blink waveforms are shown in figure 7(A). Blink artifacts were most pronounced in the frontal channels on the scalp EEG. Baseline-to-artifact ratios confirmed that these deflections were significantly above the ongoing neural activity recorded by scalp EEG (mean 0.62 ± 0.15, permutation *p*-value 0.0002). In contrast, blink artifacts were undetectable relative to ongoing baseline activity on the stent-electrode array (mean 1.10 ± 0.11, local reference). Within this participant, the difference in blink baseline-to-artifact ratios between modalities was substantial (Δ = 0.48, Cohen’s *d* = 3.23, permutation *p*-value = 0.0002, figure 7(B)).

Given both the subclavicular location of the vECoG hardware and the intravascular placement of the stent-electrode array, we next examined the impact of cardiac-related contamination. Cardiac contamination in the neural recordings was assessed by evaluating against an ECG lead placed on the chest. The average ECG waveform, time-locked to the R-peak, was compared to the residual signals recorded with the stent-electrode array with an IRTU referencing scheme (figure 7(D)), which shows residual ECG activity, albeit much lower in amplitude compared to the external ECG lead. ECG contamination was quantified by computing the normalized similarity between the external ECG waveform and each channel of the neural recordings. vECoG recordings exhibited moderate ECG contamination with the IRTU reference (mean 0.26 ± 0.02, permutation p-value 0.00049) and minimal ECG contamination with local reference (mean 0.07 ± 0.03, permutation *p*-value 0.00098). In contrast, the scalp EEG channels showed very small but contamination above zero (mean 0.01 ± 0.01, permutation *p*-value 0.0002). Within this participant, higher ECG contamination were observed for the vECoG (IRTU ref: Δ = 0.25, Cohen’s *d* = 29.14, permutation *p*-value = 0.0002; local ref: Δ = 0.05, Cohen’s *d* = 3.74, permutation *p* = 0.0002, figure 7(E)).

Finally, we quantified line noise artifact by measuring the 60 Hz peak prominence relative to the surrounding bands. Across channels, the scalp EEG had a mean peak prominence of 5.0 ± 4.3 dB (permutation *p*-value 0.0002), while the vECoG with IRTU reference had 4.0 ± 1.2 dB (permutation *p*-value 0.0005). Utilizing the local reference in the vECoG substantially reduced this common mode noise to 1.0 ± 0.9 dB (permutation *p*-value 0.002, figure 7(G)).

### 3.6. Resting-state recordings

**Figure 8.**
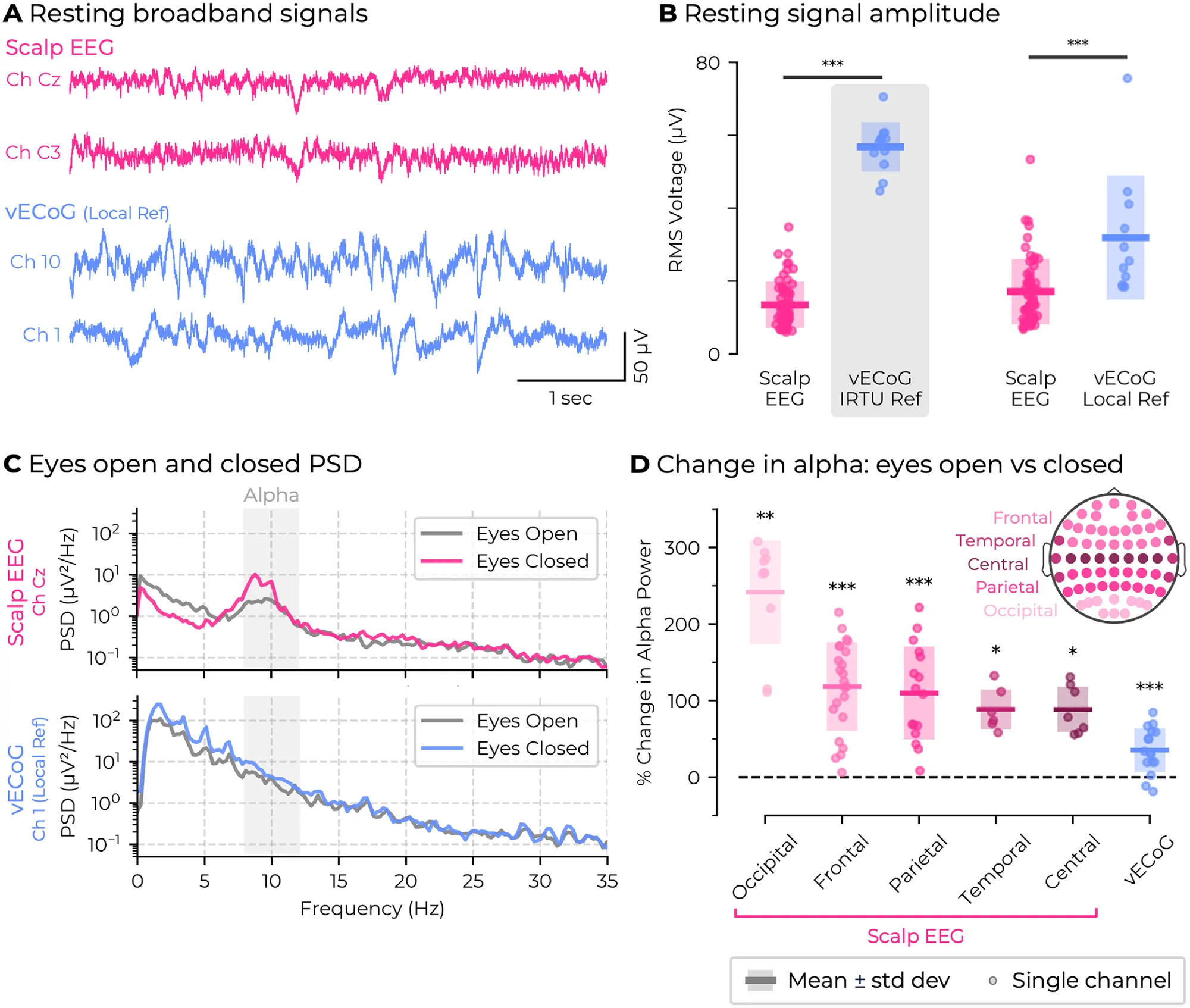
Simultaneous resting-state recordings from scalp EEG and vECoG. (A) Example broadband (highpass filtered at 1 Hz) resting-state signals from scalp EEG and vECoG recordings. (B) RMS voltage across channels, comparing stent reference (right) and IRTU reference (left) configurations on the stent-electrode array. In both cases, vECoG had a significantly larger mean amplitude (exploratory, within-subject permutation test). (C) Example power spectral density (PSD) comparing eyes-closed vs eyes-open conditions. The alpha band (8–12 Hz) is highlighted in gray. (D) The change in alpha power from eyes-open to eyes-closed for each channel, categorized by brain region. The thick line shows the mean and shading denotes 1 standard deviation. Black dashed lines indicate 0 or no change. The stars above indicate if the mean percent change in alpha power differed from zero (exploratory, within-subject one-sample permutation test). The EEG head map shows grouping of channels by brain region. Asterisks denote significance level: *p ⩽ 0.05, **p ⩽ 0.01, ***p ⩽ 0.001, and ****p ⩽ 0.0001.

Finally, we assessed baseline signal characteristics in both modalities by analyzing the resting-state recordings. Examples of resting-state recordings from scalp EEG and vECoG are shown in figure 8(A). Across channels, RMS voltage was significantly higher in the vECoG recordings (mean 31.9 ± 17.1 *μ*V) compared to scalp EEG recordings (mean 17.0 ± 8.9 *μ*V, local reference; Δ = 14.84, Cohen’s *d* = 1.42, permutation p-value = 0.0004, figure 8(B)), indicating greater baseline signal power in the intracranial recordings. vECoG recording utilizing the IRTU referencing scheme exhibited even larger RMS voltage (mean 56.78 ± 6.80 *μ*V; Δ = 43.31, Cohen’s *d* = 6.73, permutation *p* = 0.0002), likely driven by the presence of residual ECG artifact.

To determine whether eyes-open versus eyes-closed conditions significantly modulated baseline neural activity, we analyzed changes in alpha band power (8–12 Hz) across modalities. Figure 8(C) shows example power spectral densities for eyes-open and eyes-closed conditions for an example channel for each modality. Both scalp EEG and vECoG recordings exhibited increases in alpha power during eyes-closed compared to eyes-open (occipital 241%, frontal 118%, parietal 109%, temporal 89%, central 89%, and vECoG 35%, all permutation *p*-values < 0.05, figure 8(D)). The stronger alpha modulation in scalp EEG is expected given its broad spatial coverage, including occipital regions, which are known to exhibit robust increase in alpha power during eyes-closed conditions [31, 32]. In contrast, the stent-electrode array was deployed along the midline near the precentral gyrus, where alpha modulation is less pronounced, likely contributing to the relatively smaller effect.

## 4. Discussion

This study provides the first direct, within-subject comparison of neural signals recorded simultaneously from an implanted stent-electrode array and scalp EEG. To assess signal quality between scalp EEG and vECoG recordings, signal and noise characteristics were quantified during cued motor tasks and baseline resting-state conditions in one participant. Despite clear motor-related modulation in both modalities, intravascular recordings in this participant demonstrated greater motor-related modulation. Both modalities exhibited significantly reduced modulation during motor imagery relative to overt motor execution. Spatial differences, measured as a change in the centroid of modulation, between left and right ankle movement was not significantly different in this participant for either modality. Differences in artifact susceptibility appeared to reflect array location, with scalp EEG predominantly affected by superficial ocular and myogenic sources, whereas vECoG was primarily influenced by cardiac-related signals.

Only a few studies in humans have directly compared the properties of neural signals recorded simultaneously from the brain using implanted and scalp-based electrodes, especially in the context of BCI. Ball *et al* demonstrated that eye blinks artifacts were up to 100 times larger in scalp EEG than ECoG [33]. In patients with a hemicraniectomy, scalp EEG electrodes placed over the craniectomy site had significantly higher resting power and significantly greater movement-related beta and high gamma modulation compared to electrodes over the skull [6]. Given the recent development of endovascular arrays, even fewer studies have directly compared vECoG to other recording modalities. However, John *et al* reported that subdural, epidural, and endovascular ECoG all had comparable SNR of evoked potentials from median nerve stimulation in a sheep model [34].

### 4.1. Signal quality considerations

As expected, amplitudes recorded by the implanted endovascular electrodes were considerably larger than those obtained from non-invasive scalp electrodes, with an 88% larger mean voltage in vECoG compared to scalp EEG. Similarly, consistent with the closer proximity to the neural source, motor-related modulation was generally greater with vECoG compared to scalp EEG. Notably, in this study we intentionally limited preprocessing to quantify the baseline signal quality; however, there are a wide range of established signal processing techniques, such as blind source separation, which would be expected to enhance the signal quality for both vECoG and scalp EEG [35, 36]. Accordingly, the differences in signal quality observed should be interpreted as conservative estimates of the relative capabilities of each modality and not an upper bound.

For practical BCI use, ideal control extends beyond binary on-off to enable richer, more flexible control. Accordingly, we examined spatial lateralization and differentiation of motor effort as complementary approaches to increase the controllable neural dimensions. Lateralized movements (e.g. left vs right) engage prominently contralateral cortical regions, which may generate distinct spatial activation patterns that can be resolved. However, although the participant successfully performed unilateral movement and fMRI activity demonstrated lateralized activity, only modest differences were observed in the neural activity patterns. This limited differentiation is likely influenced by the spatially widespread nature of low-frequency activity, such as the alpha and beta bands, which may lack the spatial resolution necessary to resolve these patterns, especially for EEG in lower limb movements whose cortical representations lie near the midline [15, 26, 28, 36, 37]. Given its close proximity to the medial wall of both motor cortical hemispheres, stent-electrode arrays may provide modest advantages for differentiating left and right lower-limb movement, although this requires further validation. For both modalities, differentiation between left and right hemispheric activity may improve with more trials or spatial referencing and feature extraction approaches, such as Laplacian filtering or common spatial patterns [38, 39].

In agreement with prior literature, event-related desynchronization was substantially reduced during motor imagery compared to motor execution in both scalp EEG and vECoG, with 51% and 43% modulation, respectively [13, 40]. As expected in the lower-frequency bands, overt and light ankle movement exhibited similar levels of modulation, potentially reflecting contributions from somatosensory input associated with physical movement [41]. Overall, the changes in modulation across the different effort levels followed similar patterns between scalp EEG and vECoG, suggesting similar neural features recorded across both modalities.

Unexpectedly, vECoG recordings did not demonstrate significant modulation in the high gamma band (65–200 Hz), despite this frequency range typically showing robust task-related changes in invasive recordings, particularly during motor execution [11, 13, 15]. This finding was surprising given that the movements performed in this study involved overt motor execution. Prior work has demonstrated that high gamma activity can be detected using stent-electrode arrays, suggesting that the absence of modulation in the present dataset is unlikely to reflect a fundamental limitation of the recording modality [16, 17]. Instead, it may be related to specific experimental factors, such as task design, movement type, or electrode positioning. Additionally, high gamma synchronization is known to be more spatially focal than lower-frequency activity such as beta band desynchronization [15], which may have limited its detectability under the chosen recording conditions. However, the precise reason for the lack of high gamma modulation under the current recording conditions remains unclear and warrants further investigation.

### 4.2. Noise and artifact considerations

To provide a comprehensive characterization of signal quality across modalities, we quantified the impacts from noise and artifact separately from the neural signal, recognizing that optimal signal quality reflects both strong volitionally modulated signal and minimal noise. We focused on artifact types commonly reported in the literature, including eye movement, muscle activity, and line noise. Each modality demonstrates vulnerability to specific artifacts, consistent with its anatomical placement and prior literature. In particular, the scalp EEG showed high susceptibility to noise from eye blinks and scalp and facial muscle contractions, which has been well documented given its proximity to superficial physiological sources [42, 43]. Specifically, frontalis and temporalis muscle activity produce large broadband power increases in the beta and gamma bands in scalp EEG. This activity is commonly caused by facial expression such as raising the eyebrows, jaw clenching, or chewing, and can mask underlying neural signals [29, 30, 42, 44, 45]. Similarly, ocular artifacts such as blinks produce stereotyped, large transient deflections in the frontal electrodes [33, 46], which has also been reported in fronto-anterior ECoG electrodes [33]. In contrast, vECoG exhibited cardiac activity, which may be attributable to the IRTU located in the subclavicular space, as reported with other neurotechnologies [47]. Although local referencing reduced the ECG contamination, residual artifact remained present, suggesting that ECG contamination in vECoG recordings may still be an important consideration for analyses and decoding.

Importantly, the aim of this study was not to develop or optimize artifact removal methods, as this has been addressed extensively in the literature [35, 48], but rather to quantify their presence and relative impact in each modality. Indeed, many artifact types that were systemically characterized are of limited concern for decoding. For example, 60 Hz line noise appears in narrowband peaks which is easily removed with standard notch filtering, and ECG primarily affects low frequencies below 40 Hz [49, 50] and thus sparing the gamma bands. Moreover, signal processing tools, such as independent component analysis or adaptive filtering, may provide additional reduction in ECG contamination [49, 51].

Importantly, prior to quantifying the signal quality and noise characteristics, post-processing was implemented to remove artifactual or bad trials. Because our goal was to characterize the true neural signal characteristics as accurately as possible, the approach represents an idealized recording condition. Bad trials were defined by large amplitude activity or transients that affected multiple electrodes simultaneously. Overall, 5% of trials were excluded, with the majority attributable to scalp EEG (4.75%) and only a small fraction vECoG (0.25%; supplemental table 1). When this post-processing step was omitted, (keeping in just one contaminated trial) resulted in a modest but significant reduction in signal quality in some frequency bands, measured as Euclidean distance between rest and move conditions (supplemental figure 1). While this post-processing procedure improves offline signal characterization, using visual inspection is not compatible with online control. Similar correction could be incorporated into an online pipeline, however, doing so would require an additional real-time artifact detection and correction stage (e.g. automated blanking or source separation approaches). Without rejection, this artifactual activity may produce false positives or, if rejected online, would likely prevent the participant from generating a valid command for some time. The markedly lower incidence of bad trials in the vECoG recordings therefore represents a practical advantage for prospective online control.

Of note, all data for this study was collected in the participant’s home, without laboratory shielding and with the presence of external environmental distractions such as people, pets, and technology. This home use context reflects the conditions under which BCI will need to operate in daily life as a viable clinical neuroprosthetic. Overall, many of the artifacts should be viewed primarily as engineering challenges rather than fundamental limitations of the recording modality. Hardware refinements, improved shielding, optimized referencing, and tailored signal processing is likely to mitigate or eliminate much of the contamination observed.

Importantly, the relevance of artifacts may change as degenerative diseases progress. For example, as ALS advances and paralysis becomes severe, activity from head, facial, and ocular muscles diminishes, thereby reducing the impact of these artifact sources. Conversely, disease progression may introduce new sources of noise, such as ventilators or other medical devices.

### 4.3. Limitations

This study has several limitations. First, this study is limited to one participant and only two recording sessions. Importantly, the participant (C6) had previously demonstrated accurate and reliable BCI performance with the endovascular BCI with attempted right hand movement as their primary control strategy. In contrast, the lower limb movements in this study reflected motor execution rather than motor attempt. Ankle movements were selected in part because they enabled objective confirmation of overt movement versus motor imagery; however, this represents a tradeoff of experimental validity and ecological validity, as motor execution is not representative of many intended users. Further studies are needed to determine whether observed findings generalize to motor attempt, particularly in individuals with more severe paralysis.

As this is a single-participant case study, further studies are needed to verify the generalizability of these findings across a larger sample of participants and examine possible etiological differences. The present two-session dataset reflects practical constraints imposed by other experimental priorities, including ongoing BCI training. Replication of these findings across additional recording sessions would strengthen confidence in the observed differences between modalities. Long-term longitudinal sampling would further improve our understanding of the day-to-day variability and stability differences between modalities. Second, the experimental design was offline with no feedback, which restricts real-time learning and neural adaptation and prevents evaluation of online performance. Third, the scalp EEG utilized a standard, commercially available 64-channel cap; higher density and more advanced hardware may improve relative performance, while lower channel count or dry electrode systems that offer more real-world practicality may reduce signal quality. As technological advances continue, next-generation stent-electrode arrays will likely improve signal properties and performance as well.

Given the signal properties of scalp EEG are well characterized, the analysis concentrated on features more relevant to vECoG. Analysis focused on sensorimotor rhythms, over alternative control signals such as steady-state visual evoked potentials or P300 responses which are common in EEG. Additionally, two referencing schemes were evaluated with vECoG during resting-state recordings to assess the impact on signal properties. Given the limited time available for data acquisition, testing all possible combinations of features and referencing schemes for both modalities was not feasible. Furthermore, as the participant was enrolled in a trial evaluating the endovascular BCI, in order to avoid interfering with ongoing training by introducing a decoder, the scalp EEG signal was not used as an online control signal. Nevertheless, other studies have demonstrated one degree of freedom control by utilizing movement-related low-frequency desynchronization, suggesting that the signals characterized here could support simple control paradigms [52, 53]. However, future studies should investigate how the observed differences in signal quality between scalp EEG and vECoG impact BCI performance, including classification accuracy, number of independent degrees of freedom, and usability.

## 5. Conclusion

In conclusion, we provide the first exploratory, within-subject direct comparison of vECoG and scalp EEG in a human participant. Collectively, these analyses provide a comprehensive characterization of the relative strengths and limitations of recorded signal quality across modalities in this participant. As implantable BCIs move toward clinical availability, direct comparisons such as those presented here may help inform the extent to which implanted approaches outperform non-invasive alternatives. Within this participant, the observed strong motor-related modulation and low contamination provide preliminary evidence for endovascular stent-electrode arrays to support neural signal acquisition for BCI applications.

## Data Availability

The data cannot be made publicly available upon publication because they contain commercially sensitive information. The data that support the findings of this study are available upon reasonable request from the authors. vECoG source data for this study can be requested for research purposes from the Data Archive for the Brain Initiative repository, with the identifier https://dabi.loni.usc.edu/dsi/1UG3NS120191. Supplementary data available at https://doi.org/10.1088/1741-2552/ae9eef/data1.
